# The Impact of LED Lighting Spectra in a Plant Factory on the Growth, Physiological Traits and Essential Oil Content of Lemon Balm (*Melissa officinalis*)

**DOI:** 10.3390/plants11030342

**Published:** 2022-01-27

**Authors:** Hail Z. Rihan, Naofel Aljafer, Marwa Jbara, Lynn McCallum, Sabine Lengger, Michael P. Fuller

**Affiliations:** 1 School of Biological and Marine Sciences, Faculty of Science and Engineering, University of Plymouth, Plymouth PL4 8AA, UK; naofel.aljafer@plymouth.ac.uk (N.A.); mfuller@plymouth.ac.uk (M.P.F.); 2 Phytome Life Sciences, Launceston PL15 7AB, UK; 3 School of Biomedical Sciences, Faculty of Health, University of Plymouth, Plymouth PL4 8AA, UK; marwa.jbara@plymouth.ac.uk (M.J.); lynn.mccallum@plymouth.ac.uk (L.M.); 4 School of Geography, Earth and Environmental Sciences, Faculty of Science and Engineering, University of Plymouth, Plymouth PL4 8AA, UK; sabine.lengger@plymouth.ac.uk

**Keywords:** vertical farming, 435 nm, 450 nm, white light, light quality, growth, chemical profile

## Abstract

With the recent development of LED lighting systems for plant cultivation, the use of vertical farming under controlled conditions is attracting increased attention. This study investigated the impact of a number of LED light spectra (red, blue, green and white) on the growth, development and essential oil content of lemon balm (*Melissa officinalis*), a herb and pharmaceutical plant species used across the world. White light and red-rich light spectra gave the best outputs in terms of impact on the growth and yield. For blue-rich spectra, the development and yield was lower despite having a significant impact on the photosynthesis activity, including Fv/Fm and NDVI values. For the blue-rich spectra, a peak wavelength of 450 mn was better than that of 435 nm. The results have practical value in terms of increased yield and the reduction of electricity consumption under controlled environmental conditions for the commercial production of lemon balm.

## 1. Introduction

Lemon balm belongs to the Mint family (*Lamiaceae*) and grows widely in central and southern Europe and in Asia minor. It is cultivated globally because of its culinary and medicinal properties [1]. It has important applications as a herbal treatment for stress and anxiety, and has antioxidant properties that are of use in pharmaceutical applications [2] and is used in perfumes, cosmetics, tea and food products [3]. It also has antibacterial properties and a sedative impact, and these are attributed to its flavonoid and essential oil content [4,5]. The chemical composition of lemon balm leaves also include polyphenolic compounds, such as trimeric compounds, rosmarinic acid and other flavonoids. Its leaves are used in raw form as a salad vegetable in various parts of the world [6].

Light is one of the main factors influencing the physiology, growth, development and chemical composition of plants [7]. The major impact of light in plants is on photosynthesis which utilises Photosynthetically Active Radiation (PAR) comprising wavelengths of light between 400–700 nm However, plants do not respond uniformly to all wavelengths of PAR and red (600–700 nm) and blue wavelengths (420–460 nm) are the most effective at driving photosynthesis due to the absorption capacity of the light absorbing pigments chlorophyll a and b. Other wavebands play a crucial role in photo-morphological development, especially in the far-red region (above 700 nm) and some might cause harm to plant cell DNA (below 400 nm, for example) [8].

Horticultural Light Emitting Diodes (LEDs) modules have been recently developed as artificial or supplementary grow lights [9]. They have potential for use as supplementary lighting in glasshouses and sole-source lighting options in plant factory systems, where plants are grown indoors under controlled environmental conditions [10]. LEDs have many positive features, such as linear photon output, durability and long operating lifespan, as well as a capacity for construction in large arrays that produce high PAR suitable for plant growth and development. Furthermore, LED modules emit less heat than traditional lighting systems such as High-Pressure Sodium, Halide and Fluorescent tubes [11,12,13,14,15,16]. More importantly, spectral specificity can be introduced through the design of the LED array, utilising a mixture of LEDs with different wavelengths and this can be managed through the appropriate control systems [17]. This, in turn, has a high research and commercial application, due to the fact that plant species respond differently to various wavelengths, owing to specific differences in their photoreceptors [8,18].

The impact of LEDs on the growth, shape, yield and edible quality parameters of several plant species has been the subject of an increasing amount of recent research [19,20]. Moreover, a significant amount of research has confirmed the impact of LEDs on chemical composition, such as vitamin C content, soluble sugar [21], chlorophyll content [22] and the protein level and anti-oxidant activity of several plant species [23].

That said, a certain amount of research on the impact of LEDs and light wavelength on lemon balm has been previously reported [7,24]. The authors of [7] compared the use of florescent lamps (FL) with the use of white LED lights (Philips LEDs) and they reported that light sources did not have a significant impact on the growth and yield of lemon balm plants, but these plants were featured by a higher net photosynthesis value when grown under FL lamps as compared to LEDs. It was also reported that lemon balm’s chemical composition was significantly affected by the lighting conditions; for example, lemon balm had a higher content of macro- and micronutrients when they were grown under LEDs compared with fluorescent lamps [24]. Despite the published research about the impact of light conditions on the growth and yield of lemon balm, a better understanding of the impact of wavelength on the physiology, growth, yield, essential oil content and quality is still needed. The aim of this study, therefore, is to investigate the impact of wide range of light spectra including various combinations of blue, red and green on the physiological and chemical traits of lemon balm. One of its main objectives is also the investigation of the impact of blue-light wavelength sources on the physiology, growth and quality of lemon balm, since our recently published research showed that this could have a great impact on both growth and quality of some plant species, such as basil [17].

## 2. Material and Methods

### 2.1. Plant Material

**Figure 1:**
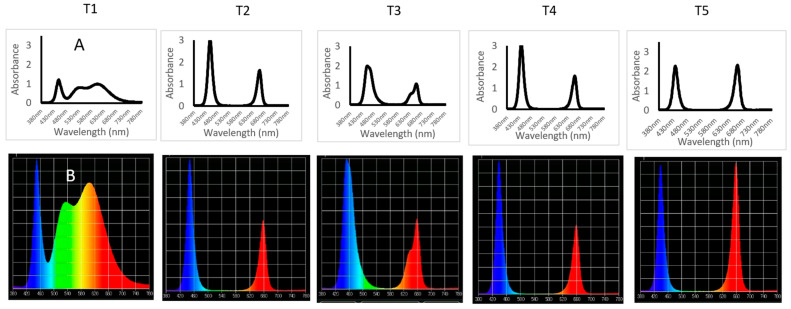
Spectra of the lighting treatments used, as measured by a UPRtek spectrophotometer: (**A**) the radiant density of the light spectrum intensity and (**B**) the relative light intensity.

**Figure 2:**
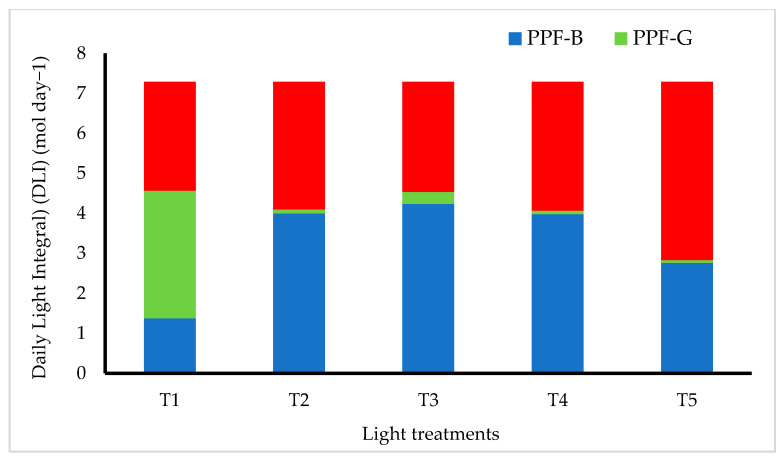
Daily light integral for different light spectra of the applied light treatments.

Lemon balm (*Melissa officinalis*) seeds were obtained from CN seeds (CN Seeds, Pymoor, Ely, Cambridgeshire, UK). Seeds were sown and germinated in Rockwool cubes (36 mm) under dark conditions and 22 ± 2 °C for 10 days, and were then transferred to an Ebb and Flow hydroponic system in the Plant Factory facility at the University of Plymouth. The Plant Factory facility is a converted insulated greenhouse, in which external light has been excluded and a multi-tier hydroponic growing system, consisting of Ebb and Flow trays with interchangeable LED light units, has been installed. The Plant Factory system is divided into several multi-shelf hydroponic units, each consisting of three tiers. The distance between tiers is 50 cm. Temperature and humidity were monitored using Gemini data loggers (Tinytag Plus (part No GP-1590)) and an instantaneous thermometer (Fisher Scientific, Loughborough, UK) at 23 ± 2 °C and humidity at 65 ± 5%. The dark/light period was set to 8/16 h. Five lighting treatments were designed and applied using LuminiGrow LED lighting systems (LuminiGrow, Shenzhen, China). The light treatments included several combinations of blue (B), green (G) and red (red) at different ratios described as follows: T1—white (B:G:R, 1:2.3:2); T2:—blue-rich with blue peak at 450 nm (B:G:R, 1:0.02:0.8); T3—blue-rich with blue at 450 nm + green (B:G:R, 1:0.07:0.65); T4—blue-rich with blue at 435 nm (B:G:R, 1:0.02:0.8); and T5—red-rich (B:G:R, 1:0.025:1.6) (Figure 1 and Figure 2).

Light intensity from the LED lighting treatments was measured using a UPRtek spectrophotometer (UPRtek MK350N premium Standalone handheld spectral light meter, Taiwan) and adjusted to deliver 125 ± 10 µmol m^−2^ s^−1^. Daily light integral to the LED light treatments were calculated at 7.2 mol day^−1^ (Figure 2). The emitted light spectra of the lighting treatments were measured (relative light intensity) using a UPRtek spectrophotometer and corrected to show the radiant density at each wavelength (Figure 1).

Growth and physiological responses of lemon balm to the lighting treatments were measured at the harvest stage (64 days from sowing). Growth/yield measurements including plant height (cm) (*n* = 8) and leaf area (LA cm^2^) (*n* = 8) were made, using a leaf area image analyser HITACHI KP-D40 colour digital camera with a lightbox and WinDias 1.5 software (Delta-T Devices Ltd., Cambridge, UK). Fresh weight (FW) (*n* = 4) and dry weight (DW) (g) (*n* = 4) were measured after removing the root system, using a Fisher Scientific SG-402 laboratory balance. For dry weight, plants were dried at 60 °C for 96 h [25].

### 2.2. Chlorophyll Fluorescence Imaging System (Fv/Fm and NDVI)

Fluorescence image acquisition was performed with a PSI open FluorCam FC 800-O (PSI (Photon Systems Instruments), Drasov, Czech Republic) applying a protocol derived from Méline et al. [26]. The system has four individual LED panels in two pairs. The first pair of LED panels provides an orange actinic light with an intensity up to 400 µmol m^−2^ s^−1^, and a wavelength of approximately 620 nm. A second pair of LED panels gives a saturating pulse with an intensity up to 3000 µmol m^−2^ s^−1^, with a blue wavelength around 455 nm. The system sensor is a CCD camera, which has a pixel resolution of 512 by 512 and a 12-bit dynamic. When photosynthetic yield is at zero, fluorescence emission reached a maximum (Fm). Fv, a variable fluorescence, defined as the difference Fm − F0. Fv and Fm, are used to calculate the maximum quantum yield of QY max = Fv/Fm. This was measured after 20 min of dark adaptation. Another important vegetative index called “normalized difference vegetation index” (NDVI), is based on the spectral reflectance of plants in the near infrared region (λ = 700–1300 nm) and the visible red range (λ = 550–700 nm) of the electromagnetic spectrum. Dark adaptation is 20 min and calculation of NDVI is:

NDVI = (NIR − Red)/(NIR + Red)

### 2.3. Chlorophyll Content

Chlorophyll content was evaluated, using the method described by [27]. Plant tissue (leaves) (0.2 g) was ground with 10 mL 80% acetone. The final volume was made up to 10 mL with 80% acetone and then centrifuged for 3 min. Absorbance was measured against an 80% acetone blank. Supernatant (2 mL) was placed in a cuvette and the absorbance was measured at 663.6 (A_663.6_) and 646.6 (A_646.6_), using a Jenway 7315 (Staffordshire, UK). The formulae are based on the absorbance maxima of each pigment and are dependent on the solvent used. The formulae for samples dissolved in acetone are as follows:

Ca = 12.25 A_663.6_ − 2.55 A_646.6_

Cb = 20.31 A_646.6_ − 4.91 A_663.6_

Total C = 17.76 A_646.6_ + 7.34 A_663.6_

where Ca: chlorophyll A, Cb: chlorophyll B, Total C: total chlorophyll. 

The values obtained were converted to estimate the chlorophyll content per gram of fresh weight, following the procedure described by [27].

### 2.4. Essential Oil Analysis

Leaves were collected and dried, then 10 g were ground in a mortar and pestle. The essential oil was extracted employing the Soxhlet method, using absolute ethanol (Thermo Fisher Scientific, Loughborough, UK) as a solvent [28]. Through this method, 10 g of dry lemon balm was extracted for 4 h. Once the extraction was complete, essential oil was separated from the solvent, using a BÜCHI R-124 Rotary Evaporator System (BUCHI UK Ltd., Suffolk, UK). The essential oils were then collected in a vial. The vial was weighed before and after the extraction to calculate the quantity of the essential oil obtained.

### 2.5. Statistical Analysis

The main experiment consisted of 5 lighting treatments with 4 replicates, each consisting of 15 plants. Treatments were randomised at each replication. Results were presented as means ± standard error (S.E.). All data were subjected to analysis of variance (ANOVA) using Minitab software (version 19). Tukey’ post hoc test was used for determination of significant differences between the treatments. Comparisons of means were made using the least significant difference (LSD) test at a 95% level of probability.

## 3. Results

### 3.1. Growth/Yield Responses to LED Light Treatments

Although all lighting treatments produced plants of an acceptable commercial quality, there was a significant impact of different spectra on various growth and yield parameters.

**Figure 3:**
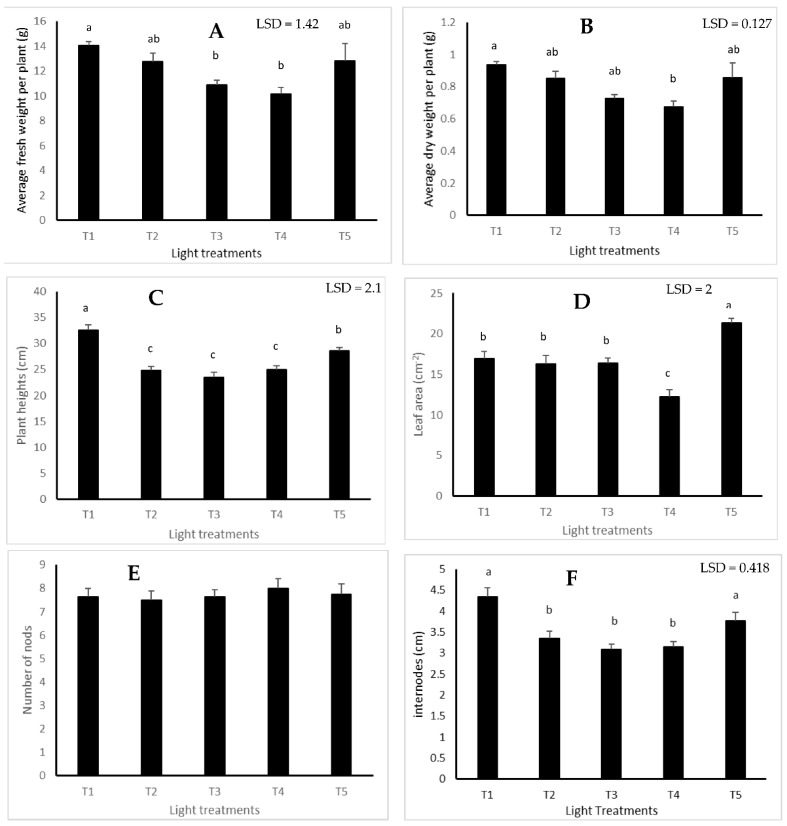
The effect of light treatment on growth traits: (**A**) fresh weight (g); (**B**) dry weight (g); (**C**) plant heights (cm); (**D**) leaf area; (**E**) number of nodes; (**F**) internodes (cm) of lemon balm measured at the harvest stage (64 days from sowing) (Means denoted by a different letter indicate significant differences between treatments (*p* < 0.05)).

White light (T1), which is the only spectrum that included a high level of green wavelengths in addition to red and blue, had a significant impact of the average fresh weight (*p* = 0.004), average dry weight (*p* = 0.018), plant height (*p* ≤ 0.001) and internodes (*p* ≤ 0.001) in comparison with other light treatments (Figure 3A–C,F). Red-rich treatment (T5) also seemed to have a positive impact on plant height and internodes in comparison with other treatments, apart from the white light treatment (Figure 3C,F).

Red-rich light treatment (T5) had a significant positive impact on leaf area compared with other light spectra (*p* ≤ 0.001). Light spectrum did not significantly impact the number nodes (*p* = 0.918) (Figure 3E).

With regards to the impact of blue light wavelength (T2 and T4), using blue light at 450 nm (T2) produced larger plants (fresh and dry weights) and bigger leaf area compared to the use of 435 nm (T4) (Figure 3A,B,D respectively). However, no significant impact of the blue source was observed on plant height (Figure 3C).

### 3.2. Physiological and Chemical Responses to LED Light Treatments

#### 3.2.1. Fv/Fm Ratio and NDVI Indicators

**Figure 4:**
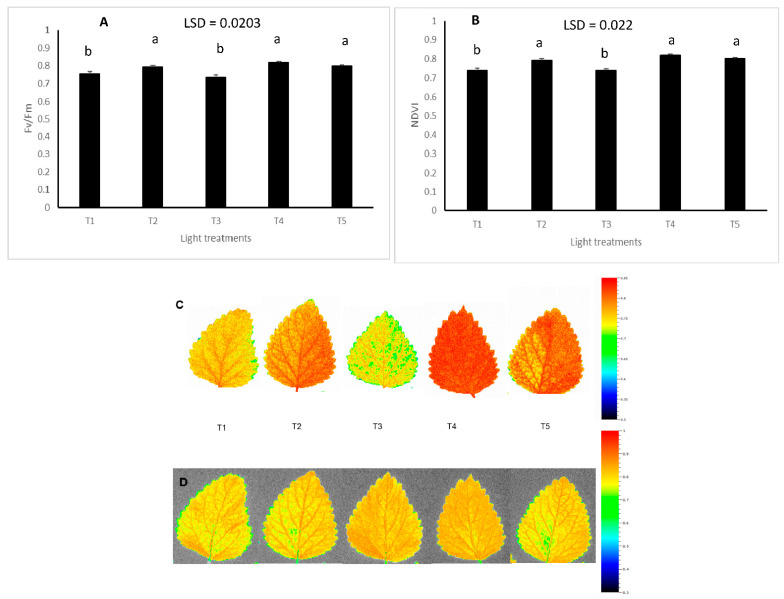
The effect of light treatments on Fv/Fm (**A**,**C**) and NDVI (**B**,**D**) (Means denoted by a different letter indicate significant differences between treatments (*p* < 0.05)).

Light treatments had a significant impact on Fv/Fm value (*p* = 0.016) and NDVI (*p* = 0.024) (Figure 4). Blue-rich spectrum with 435 nm used as a source of blue light had a significant impact on both Fv/Fm and NDVI. White spectrum (T1) and blue/red treatment that included some green in the spectrum combination (T3) had a negative impact on both Fv/Fm and NDVI (Figure 4).

#### 3.2.2. Chlorophyll Content

**Figure 5:**
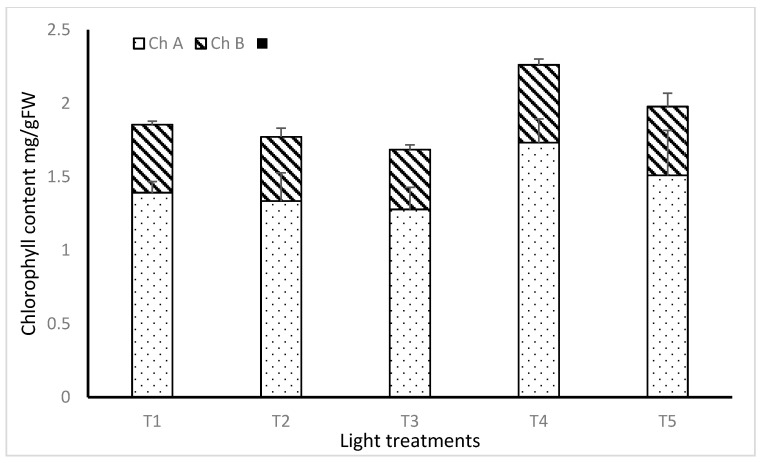
The effect of light treatment on chlorophyll content of lemon balm.

There were only small and non-significant differences in chlorophyll content among the lighting treatments either for chlorophyll A, chlorophyll B or total chlorophyll (*p* = 0.227, *p* = 0.620 and *p* = 0.315 respectively) (Figure 5).

### 3.3. The Impact of Light Treatment on the Essential Oil Content in Lemon Balm

**Figure 6:**
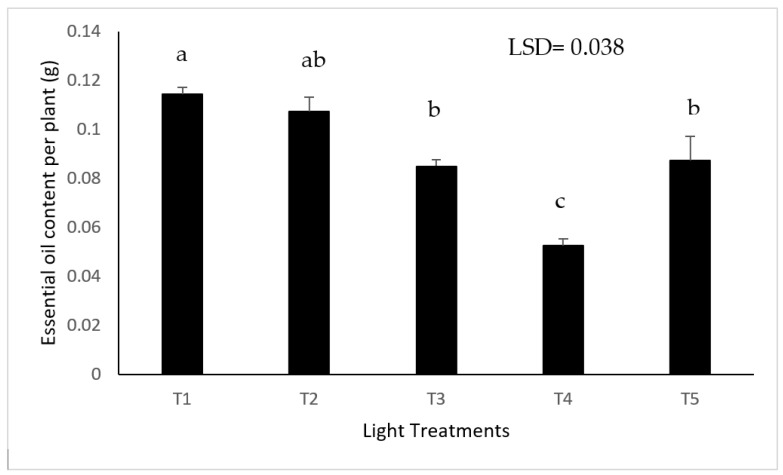
The effect of light treatment on essential oil content per lemon balm plant (Means denoted by different letters indicate significant differences between treatments (*p* < 0.05)).

Light treatment had a significant impact on essential oil content per plant of lemon balm. White light treatment had a significant impact on the essential oil yield of lemon balm in comparison with other light treatments (*p* ≤ 0.001) (Figure 6). Moreover, Blue-rich with blue at 435 nm (T4) had a negative impact on the essential oil content.

## 4. Discussion

*Melissa officinalis* (lemon balm) is an important source of active chemicals, such as triterpenes, flavonols, phenolic acid and many other important pharmaceutical compounds [29]. Similarly to other pharmaceutical plant species, the growth, yield and chemical compositions of these species are affected by environmental factors when grown under open-field conditions. However, with the recent fast development of LED grow lighting systems and the increasing efficacy of these systems, growing pharmaceutical plants, such as lemon balm, vertically under controlled environmental conditions is now viable and has potential commercial value. Vertical farming (plant factory) is a novel plant-production system that allows local production of high-quality pharmaceutical plant species [30]. In the plant factory system, LEDs are used as the sole source of lighting and provide a unique tool for promoting growth, yield and quality. However, plant species respond differently to lighting conditions and therefore it is crucial to vary light spectra and intensity to suit the requirements of individual plant species [4,17,31].

In terms of lemon balm, white (50% cool white + 50% warm light) light improved the growth traits, including fresh and dry weight, plant heights and internode spacing. This highlights the great impact of green spectrum on the growth of this plant species, since white light has a significant amount of green, which is higher than both the blue and the red spectra. This also could be due to the impact of other wavelengths such as orange, yellow, etc., which existed in the full spectrum of white and did not exist in the other described treatments in this research. This finding is in accord with other research indicating the positive impact of white light on the growth and development of plants, even by comparison with blue light added to red LEDs [32].

Kim et al. [20] reported that lettuce plants grown with spectra that included green light had better growth levels, including fresh and dry weights, than those grown with red/blue only. However, the current findings disagree with those of [10,17] which had indicated the positive impact on the development and growth of basil of focusing light in the red and blue regions. The observed differences could, however, be due to the differing responses to light of the two plant species.

The current finding showed a high positive impact of red-rich light spectra on the growth parameters of lemon balm, including fresh and dry weight, height and leaf area. Lin, Huang and Hsu [33] reported a significant positive impact of high level red light on the growth and development of green and purple basil plants. Red light is one of the essential components in lighting spectra for plant growth and red light alone is sufficient for normal plant growth and photosynthesis [34]. The current results are also in accord with what has been reported of the yield reduction associated with a high level of blue light in light spectra, a phenomenon that had been linked previously with lower internode length and smaller leaf area [32,35], and which was also observed in the current study. It was reported that red light matches the assimilation peak of the photoreceptors phytochrome and chlorophyll and that the combination of red–blue light for growing plants causes a greater improvement in the maximum photosynthetic rate than monochromatic light, as a consequence of the activation of cryptochromes, phytochromes and chlorophyll [36]. However, the current findings do not agree with what was reported by [4,17] concerning the significant impact of high-level blue light compared with red light in the light spectrum on the growth and development of basil. These conflicting results could be attributed to a difference in experimental conditions, such as the light intensity, the temperature and the plant species that was studied. One of the main challenges to the replicability of research results in LED lighting applications could be caused by the high variability of experimental setups [17,32]. For example, while 125 μmol m^−2^ s^−1^ PPFD was applied in the current research, high PPFD values, e.g., 300 μmol m^−2^ s^−1^, were applied by [17].

The use of 435 nm as a source of blue light instead of 450 nm, which is widely used in commercial undertakings, had a negative impact on the yield parameter of lemon balm. This contrasts with what was reported by [17] regarding the significant positive impact of this wavelength on the growth and development of basil. However, a significant number of research studies have reported the role of blue at 450 nm on the growth and development of plants [32]. These differences could be due to the genetic, physiological differences between plant species. Further research on the impact of blue light variations on plants is necessary.

Light spectra did not have a significant impact on the chlorophyll content of lemon balm. It is possible that the level of chlorophyll was not affected by the light treatment because all the treatment contained a sufficient level of blue and red in the spectrum combinations. It has previously been reported that LED light supplying RB increased the total amount of chlorophyll in Chinese cabbage leaves, compared with the concentration of chlorophyll in plants treated with blue or red light only [37,38]. Chen et al. [35] found that the chlorophyll content of lettuce leaves was higher when plants were grown under a mixture of red and blue spectra, compared to growth with blue or red light only.

The impact of light spectra on the photosynthesis activity of lemon balm was evaluated using a Chlorophyll Fluorescence Imaging system. Chlorophyll fluorescence imaging is an extremely important technique for the non-invasive study of photosynthesis dynamics in intact plants, algae and in cyanobacteria for the measurement of chlorophyll fluorescence kinetics. This device/technique was used to calculate the maximum quantum yield of QY max = Fv/Fm. An interesting finding of the current study was that blue light at 435 nm has a significant positive impact on both Fv/Fm and NDVI indicators compared to other light treatments. Moreover, the use of 435 nm as a source of blue has a significant impact on these indicators as compared to the same treatment with 450 nm used as a source of blue. This finding agrees with that of Rihan et al. (2020) on the significant impact of 435 nm wavelength compared with 450 nm wavelength in terms of its effects on the photosynthesis activity of basil. The 435 nm treatment had a positive impact on the stimulation of PS I in the photosynthesis process in *Cyanobacteria Bacteria* and *Arabidopsis thaliana* [39]. This could explain the significant increase in the Chlorophyll Fluorescence Rate (Fv/Fm) and the NDVI indicator observed in the current research. A fluorescence spectral analysis showed that Chamomile pollen reaches a peak in a blue light region of 435 nm [40]. However, in the current study, the significant photosynthesis activities did not translate into an improvement in the growth rate of lemon balm. There could be several reasons for this, including differences in the experimental conditions, such as light intensity, temperature, etc. More research is needed for a further understanding of the conflicting findings with regard to the photosynthesis parameters and growth traits observed in this plant species.

Although no significant impact of light spectrum on the content of essential oil was observed, there was a clear negative impact of blue 435 nm on the essential oil content. However, further studies of the impact of light spectra on the quality and chemical composition of lemon balm oil are needed.

## 5. Conclusions

Between a wide range of light spectra, including white, red/blue in various ratios and blue at different wavelengths, the best results in terms of the impact of light spectra on growth and yield were obtained using white light (50% cool white + 50% warm white). This has a high practical application, as white light has wide commercial availability and is user friendly. Moreover, blue light sources seem to have a significant impact on the growth and physiology of lemon balm. While blue at 450 nm promoted growth and increased the yield, blue at 435 nm had a significant impact on the photosynthesis activities.

## Data Availability

Data is contained within the article.
